# A Word to the South; a Thought for the North

**Published:** 1897-04

**Authors:** 


### A WORD TO THE SOUTH—A THOUGHT FOR THE NORTH.

Nashville, the mecca of 1897, is now before us, and the time for completion of all the details of our programme and convention will be all too short to make out of our gathering the most that should result from our pilgrimage to Southern climes. While we shall enjoy every hospitable privilege and courtesy afforded us by our Southern friends and colleagues, there must be borne in mind that our chief purpose in turning our faces southward from every portion of North America is to make, above all, an opening era of progress in this new and promising field. Every line possible must be thrown out to secure all the advantages within reach in adding to the measure of recognition and support of our fellow-practitioners in that territory; and the good influences exercised by our Association in other sections of our country must be redoubled there, or our mission will be only half accomplished. The present scattered locations of the Southern veterinarians will make their labors hard to perform, and the members from the North will bear this in mind. The latter will, therefore, appreciate the fact that this imposes upon them an extra duty of aiding in every way and seconding every effort promptly and to the uttermost, when solicited by their Southern colleagues, and thus assure them that our chief purpose and aim will be to make doubly sure the position of our profession in the forefront of the progress of the New South. Bear in mind that Nashville is not situated as are Buffalo, Boston, and Philadelphia, and that our sojourn there in 1897 will be preparatory for a future meeting still further south, as were the early meetings in Boston and Philadelphia. Let everyone give something as a contribution to this meeting, to the programme, to our ever-zealous, hard-working Secretary, to our Southern colleagues, to our adopted vocation, and Nashville will prove the open door to an unlimited field for the future votaries of our profession.

ON TO NASHVILLE!

